# Hydrogen Sulfide Alleviates Manganese Stress in *Arabidopsis*

**DOI:** 10.3390/ijms23095046

**Published:** 2022-05-02

**Authors:** Lixia Hou, Zhaoxia Wang, Guangxia Gong, Ying Zhu, Qing Ye, Songchong Lu, Xin Liu

**Affiliations:** Key Lab of Plant Biotechnology in University of Shandong Province, College of Life Science, Qingdao Agricultural University, Qingdao 266109, China; houlixia78@163.com (L.H.); 15771397276@163.com (Z.W.); gong286681@163.com (G.G.); zhuying0505@163.com (Y.Z.); 18500058569@126.com (Q.Y.); fudanlsc@126.com (S.L.)

**Keywords:** *Arabidopsis*, hydrogen sulfide, manganese stress, L-cysteine desulfhydrase, antioxidant enzyme

## Abstract

Hydrogen sulfide (H_2_S) has been shown to participate in various stress responses in plants, including drought, salinity, extreme temperatures, osmotic stress, and heavy metal stress. Manganese (Mn), as a necessary nutrient for plant growth, plays an important role in photosynthesis, growth, development, and enzymatic activation of plants. However, excessive Mn^2+^ in the soil can critically affect plant growth, particularly in acidic soil. In this study, the model plant *Arabidopsis thaliana* was used to explore the mechanism of H_2_S participation and alleviation of Mn stress. First, using wild-type *Arabidopsis* with excessive Mn^2+^ treatment, the following factors were increased: H_2_S content, the main H_2_S synthetase L-cysteine desulfhydrase enzyme (AtLCD) activity, and the expression level of the *AtLCD* gene. Further, using the wild-type, *AtLCD* deletion mutant (*lcd*) and overexpression lines (*OE5* and *OE32*) as materials, the phenotype of *Arabidopsis* seedlings was observed by exogenous application of hydrogen sulfide donor sodium hydrosulfide (NaHS) and scavenger hypotaurine (HT) under excessive Mn^2+^ treatment. The results showed that NaHS can significantly alleviate the stress caused by Mn^2+^, whereas HT aggravates this stress. The *lcd* mutant is more sensitive to Mn stress than the wild type, and the overexpression lines are more resistant. Moreover, the mechanism of H_2_S alleviating Mn stress was determined. The Mn^2+^ content and the expression of the Mn transporter gene in the mutant were significantly higher than those of the wild-type and overexpression lines. The accumulation of reactive oxygen species was significantly reduced in NaHS-treated *Arabidopsis* seedlings and *AtLCD* overexpression lines, and the activities of various antioxidant enzymes (SOD, POD, CAT, APX) also significantly increased. In summary, H_2_S is involved in the response of *Arabidopsis* to Mn stress and may alleviate the inhibition of Mn stress on *Arabidopsis* seedling growth by reducing Mn^2+^ content, reducing reactive oxygen species content, and enhancing antioxidant enzyme activity. This study provides an important basis for further study of plant resistance to heavy metal stress.

## 1. Introduction

As a necessary trace element for plant growth, manganese (Mn) is mainly absorbed by plants in the form of divalent Mn ion (Mn^2+^), which plays an important role in plant growth, development, and metabolism [1]. However, when the concentration of Mn^2+^ in the soil exceeds a certain threshold, it will be toxic to the plant, thus affecting normal growth. Mn stress usually occurs in acidic soil. When the pH value of the soil is lower than 5.5, a large amount of soluble Mn^2+^ is released. In these conditions, the concentration of Mn^2+^ in the soil increases sharply, which leads to Mn^2+^ accumulation in the plant [2]. With rapid industrialization and changes in tillage methods, the area of acidic soils in the world has expanded. Mn stress has become the second-largest plant-growth-limiting factor, after aluminum toxicity [3]. Therefore, it is of great significance to explore the mechanisms of Mn stress affecting plant growth and how to alleviate it.

When subjected to Mn stress, plants will behave differently at different growth stages [4]. Overall, Mn stress has a greater effect on the above-ground part than on the root system, and the leaf is the main target of Mn. Nearly 90% of the Mn absorbed by the plant is transferred to the above-ground tissue. Excess Mn^2+^ can inhibit leaf photosynthesis [5]. Mn stress decreases the activity of many important enzymes in plants [6], in addition to affecting the absorption, transport, and distribution of other nutrients that destroy the root structure [7]. In plants, Mn^2+^ is absorbed and transported through Mn^2+^ transporters. Most of these transporters are transmembrane proteins, which can transport and store Mn^2+^ in the inner membrane organelles [8,9]. However, the mechanism of how this process is initiated by plants has not fully been revealed.

H_2_S is an important gas signal molecule. Studies on endogenous H_2_S in plants date back decades. Wilson et al. (1978) observed that the leaves of cucumber, corn, and soybeans can release H_2_S [10]. Rennenberg et al. (1987) found that in *Arabidopsis*, L-cysteine desulfhydrase and D-cysteine desulfhydrase used L/D-cysteine as a substrate to produce H_2_S. L/D-cysteine desulfhydrase is a key enzyme for H_2_S synthesis in plants, and it is also the most studied H_2_S synthetase [11]. In recent years, it has been found that H_2_S can participate in plant growth, development, and metabolism, such as enhancing plant photosynthesis, delaying flowering and senescence, and promoting seed germination [12,13,14]. Additionally, H_2_S can increase plant resistance to a variety of environmental stresses, including drought, high salt, extreme temperatures, and various heavy metal stresses such as chromium [15,16], cadmium [17], and aluminum [18]. It has been reported that H_2_S can alleviate aluminum toxicity by reducing the absorption of Al^3+^ and increasing the antioxidant capacity in barley [19]. H_2_S can regulate the AsA–GSH cycle and alleviate As toxicity to peas [20]. It can also alleviate the inhibition of Cr^6+^ on the roots of *Arabidopsis* by upregulating the heavy metal (HM) chelator synthase-encoding genes, such as *PCS1*, *PCS2*, and *MT2A. Increased content of metallothionein and phytochelatins increases* *Arabidopsis* tolerance to Cr stress [21]. However, no reports have been made on H_2_S response to Mn stress.

Using wild-type *Arabidopsis*, the AtLCD defective mutant *lcd*, and the AtLCD over-expression lines *OELCD* (*OE5* and *OE32*) as materials, we performed physiological and biochemical methods to explore the function and mechanism of H_2_S in response to Mn stress in *Arabidopsis*.

## 2. Results

### 2.1. Effects of Mn Stress on H_2_S Content, AtLCD Enzyme Activity, and Gene Expression in Arabidopsis Seedlings

**Figure 1:**
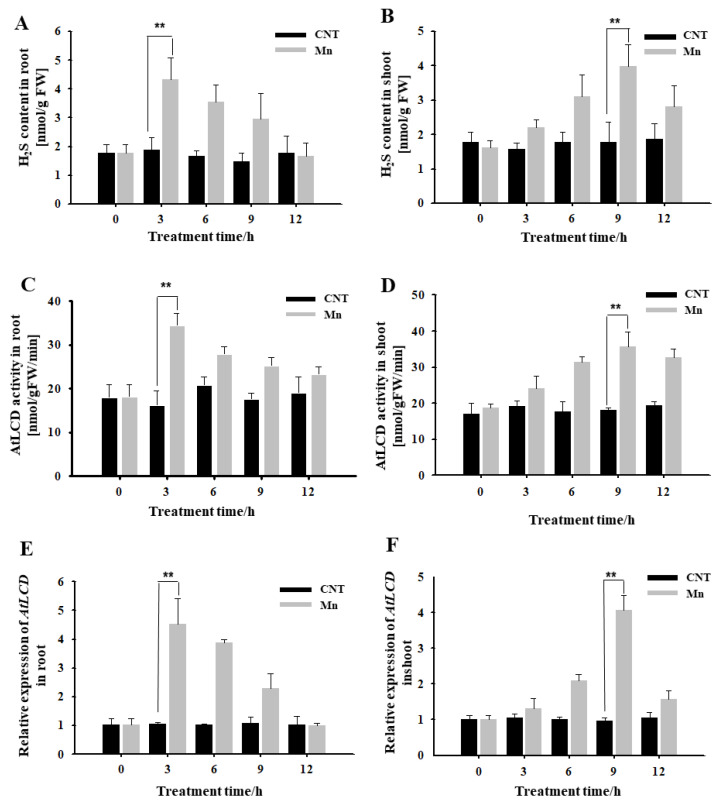
Effects of Mn stress on H_2_S content, AtLCD enzyme activity, and gene expression in *Arabidopsis* seedlings. Effects of Mn stress on H_2_S content in *Arabidopsis* roots (**A**) and shoot (**B**); AtLCD enzyme activity in *Arabidopsis* roots (**C**) and shoot (**D**); the relative expression of *AtLCD* in *Arabidopsis* roots (**E**) and shoot (**F**). Three independent experimental replications were conducted. Values are the means ± SE of three independent experiments (** *p* < 0.01).

It can be seen from Figure 1 that, under hydroponic conditions, H_2_S content in roots treated using 4 mM Mn^2+^ for 3 h increased significantly, reached a high level, and then gradually decreased (Figure 1A). Both the activity of the H_2_S synthase AtLCD and the expression of *AtLCD* reached maximum levels at 3 h and then decreased (Figure 1C,E). The H_2_S-related indicators of the shoots were tested. It was also found that Mn^2+^ caused the H_2_S content to increase, reaching the highest level at 9 h (Figure 1B). The main synthase activity and *AtLCD* expression had the same trend (Figure 1D,F). Thus, it is speculated that H_2_S may participate in the response to Mn stress.

### 2.2. Effects of H_2_S on Phenotype and Growth of Arabidopsis under Mn Stress

**Figure 2:**
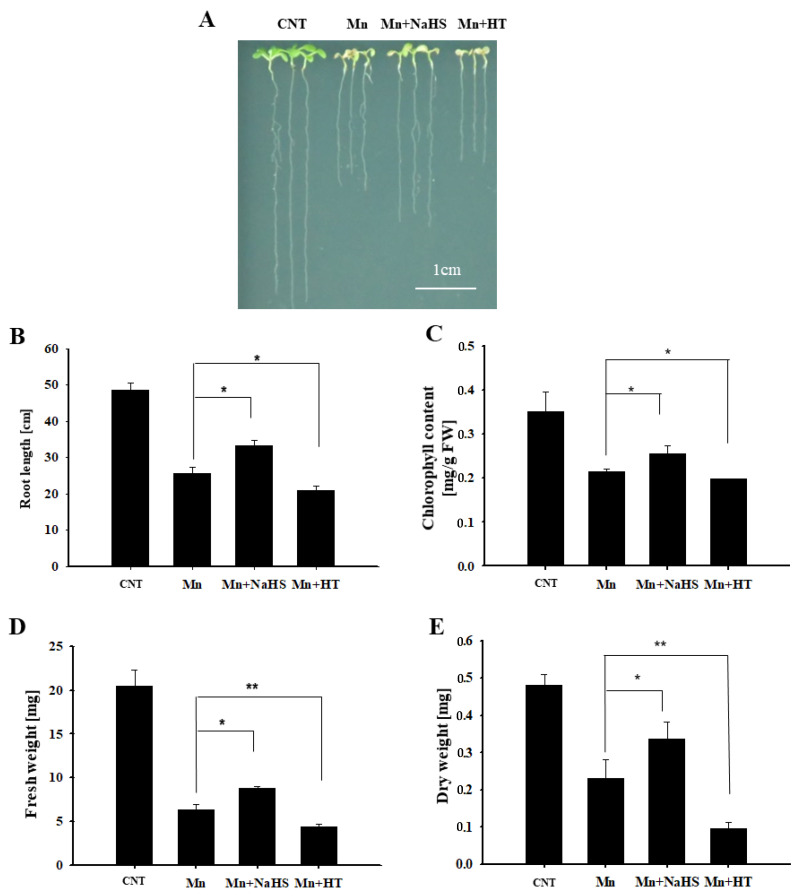
Effects of NaHS and HT on the growth of wild-type *Arabidopsis* seedlings under Mn stress. Effects of NaHS and HT on the phenotype (**A**), root length (**B**), chlorophyll content (**C**), fresh weight (**D**), and dry weight (**E**) of wild-type *Arabidopsis* under Mn stress. Three independent experimental replications were conducted. Values are the means ± SE of three independent experiments (* *p* < 0.05, ** *p* < 0.01). Scale bar = 1 cm.

To investigate the role of H_2_S in Mn stress, using wild-type *Arabidopsis* as the material, the seedling phenotype and growth indicators were observed by exogenous application of H_2_S donor NaHS and scavenger HT under 4 mM Mn^2+^ treatment after 5 d. Compared with the control, it can be seen from Figure 2A that the growth of *Arabidopsis* seedlings was inhibited by Mn^2+^ treatment, the phenotype of Mn stress was significantly alleviated after NaHS treatment, and the phenotype of Mn stress was aggravated after HT treatment. Furthermore, the main root length, chlorophyll content, fresh weight, and dry weight were calculated, and the results were consistent with the phenotype results (Figure 2B,E). We also measured whether NaHS and HT affected H_2_S content under Mn stress. The results showed that H_2_S content increased after 5 days of Mn stress; moreover, NaHS could further enhance the content of H_2_S, while HT decreased H_2_S concentration (Figure S2). This further suggests the participation of H_2_S in response to Mn stress, i.e., alleviating the stress caused by Mn^2 +^ to *Arabidopsis* seedlings.

### 2.3. Effects on the Phenotype of lcd and OELCD Lines under Mn Stress

**Figure 3:**
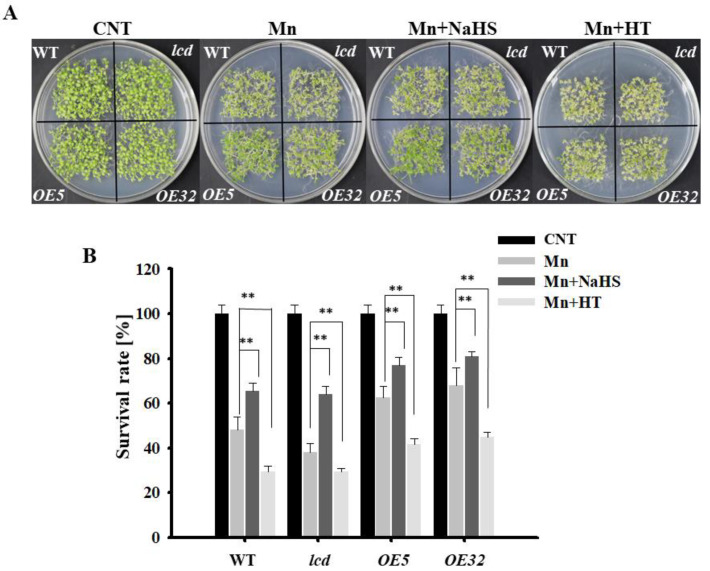
Effects of NaHS and HT on the growth of *lcd* and *OELCD* under Mn stress. Effects of NaHS and HT on the phenotype (**A**) and survival rates (**B**) of wild type, *lcd*, and two independent overexpression *Arabidopsis* seedlings after Mn stress. Three independent experimental replications were conducted. Values are the means ± SE of three independent experiments (** *p* < 0.01).

To provide genetic evidence of H_2_S participation in Mn stress, *lcd* mutant and two lines overexpressing *AtLCD* (*OE5*, *OE32*) were obtained and identified, and the results are shown in Supplementary Data (Figure S1). The phenotype and survival of each line after Mn stress were observed. The results are shown in Figure 3A. Overall, compared with *Arabidopsis* seedlings after 2 mM Mn^2+^ application, the phenotypes of all lines of Mn stress were alleviated after NaHS treatment, whereas the growth state of *Arabidopsis* seedlings was poor, and the leaves turned yellow after HT treatment. From a single treatment, after the Mn^2+^, Mn^2+^ and NaHS treatment, and Mn^2+^ and HT treatment, the phenotypes of both overexpression lines were significantly better than those of the wild type, whereas mutants showed poor growth, and the leaves turned yellow. The survival statistics were consistent with those of the phenotype (Figure 3B).

### 2.4. Effects on Mn Transporter-Related Gene Expression in Roots of lcd and OELCD under Mn Stress

**Figure 4:**
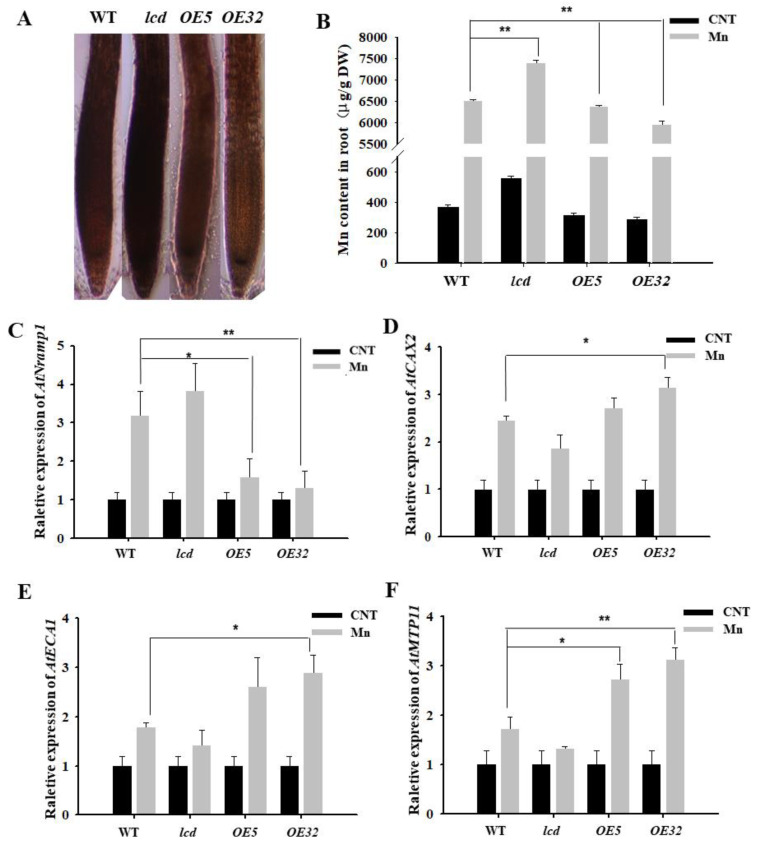
Effects on Mn transporter-related gene expression in roots of *lcd* and *OELCD* under Mn stress. Hematoxylin staining (**A**), Mn content (**B**), and the expression of *AtNramp1* (**C**), *AtCAX2* (**D**), *AtECA1* (**E**), and *AtMTP11* (**F**) in the roots of wild type, *lcd*, and two independent overexpression lines under Mn stress. Three independent experimental replications were conducted. Values are the means ± SE of three independent experiments (* *p* < 0.05; ** *p* < 0.01).

Hematoxylin is often used to observe the distribution of metal ions in plant roots [22]. Under Mn stress, the combination of hematoxylin with Mn^2+^ in root cells shows a purple color; the darker the purple color, the more Mn^2+^ in root cells. After 4 mM Mn^2+^ treatment of WT, *lcd*, and *OELCD*, hematoxylin staining was performed, and the results are shown in Figure 4A. The roots of *lcd* were stained deeper than those of the wild type, and the roots of *OE5* and *OE32* were stained shallowly. As hematoxylin staining is not specific to Mn, Mn content in roots was analyzed further by inductively coupled plasma atomic emission spectrophotometry (ICP–MS). The differences between wild type and other lines were compared. The content of Mn in *OE5* and *OE32* lines was significantly lower than that in the wild type under Mn stress, whereas, in *lcd* mutant, Mn content was significantly higher than that of the wild type (Figure 4B). It is, therefore, speculated that H_2_S may reduce the root tissue Mn content under Mn stress.

Furthermore, we analyzed whether a decrease in Mn content in root tissue by H_2_S was related to the Mn transporter. There are numerous Mn transport-related proteins in plant roots, in which AtNramp1 is located in the cell membrane and is responsible for absorbing Mn^2+^ from the external environment; AtCAX2, AtMTP11, AtECA1 are located in the endomembrane system, such as in vacuole membranes, endoplasmic omentum, and Golgi membranes, and they are responsible for transporting excess Mn^2+^ to the cell organelles when the concentration of cytoplasmic Mn^2+^ is too high, thereby alleviating Mn stress [23]. Does H_2_S reduce Mn^2+^ content in roots by regulating the expression of Mn transporter genes? The expression of Mn transporter-related genes in root tissues of WT, *lcd*, and *OELCD* was detected. The results reveal that 4 mM Mn treatments strongly induced the expression levels of four genes in all lines. The differences between wild type and other lines were further compared. The expression of the Mn^2+^-uptake-related gene *AtNramp1 in OE5* and *OE32* lines was significantly lower than that in the wild type under Mn stress, whereas, in *lcd* mutant, the gene expression level increased but did not reach a significant level (Figure 4C). At the same time, compared with wild type, the expression levels of *AtCAX2*, *AtMTP11*, and *AtECA1* in *lcd* lines exhibited reduction but not significantly, while those of *OE5* and *OE32* lines were significantly higher than that of wild type (Figure 4D,F). The expression levels of four manganese-transport-related genes changed slightly in the mutant. Therefore, it is speculated that other H_2_S synthesis genes and *AtLCD* have functional redundancy. Furthermore, it is inferred that H_2_S may have partially prevented the root intake of Mn^2+^ by reducing the gene expression of Mn^2+^-uptake-related proteins, thereby promoting the transport of Mn^2+^ to organelles by increasing partial transporter gene expression.

### 2.5. Effects on Reactive Oxygen Species Content of Arabidopsis Seedlings under Mn Stress

**Figure 5:**
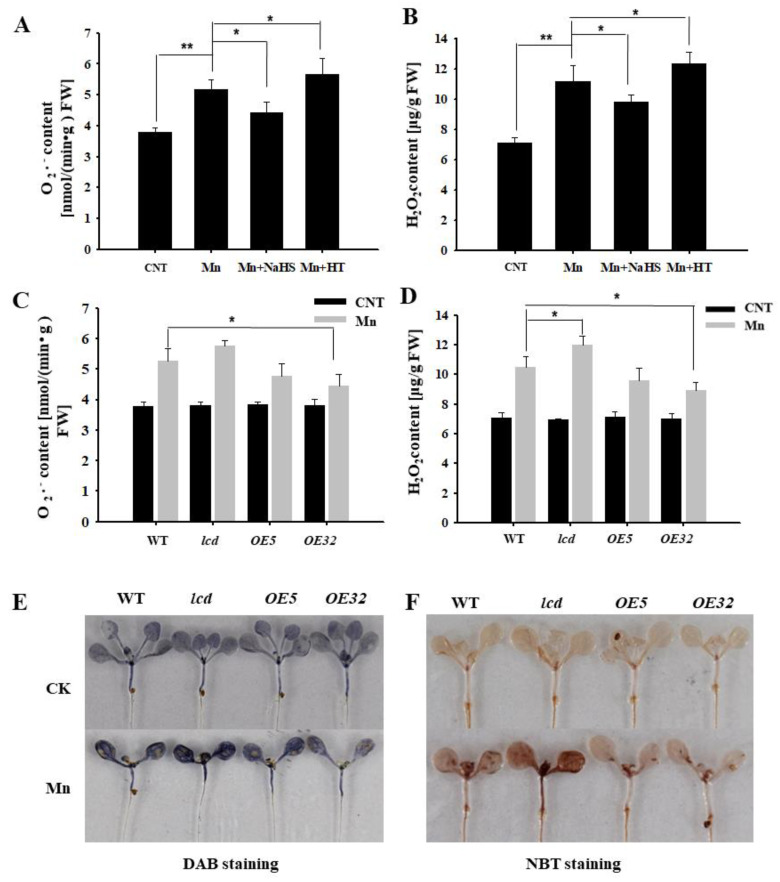
Effects of H_2_S on H_2_O_2_ and O_2_^−^ contents in *Arabidopsis* seedlings under Mn stress. Effects of NaHS and HT on the quantitative measurement of O_2_^−^ (**A**) and H_2_O_2_ (**B**) concentrations in wild-type *Arabidopsis* seedlings under Mn stress. Quantitative measurement of O_2_^−^ (**C**) and H_2_O_2_ (**D**) concentrations in wild type, *lcd*, and two independent overexpression lines seedlings treated with and without manganese. In situ accumulations of H_2_O_2_ (**E**) and O_2_^−^ (**F**) before and after Mn treatment were revealed by DAB and NBT staining, respectively. Three independent experimental replications were conducted. Values are the means ± SE of three independent experiments (* *p* < 0.05; ** *p* < 0.01).

The content of reactive oxygen species in plants increases under abiotic stresses, such as heavy metal stress. Using wild-type *Arabidopsis* as material, the content of superoxide anion and hydrogen peroxide was detected by exogenous application of NaHS and HT in 4 mM Mn^2+^ treatments. Figure 5A,B show that the concentrations of O_2_^−^ and H_2_O_2_ in *Arabidopsis* seedlings after Mn^2+^ treatment were significantly higher than that of control. The content of reactive oxygen speeies (ROS) was reduced with NaHS, compared with the Mn treatment, whereas the exogenous application of HT increased its content. Thus, it is speculated that exogenous H_2_S can reduce the reactive oxygen species in *Arabidopsis* seedlings under Mn stress.

Further evidence from genetics was provided. The concentrations of O_2_^−^ and H_2_O_2_ in *AtLCD* deficient mutants and overexpression lines were quantified under 4 mM Mn stress. The results showed that the concentrations of O_2_^−^ and H_2_O_2_ in *OELCD* lines were significantly lower than that in wild type, whereas *lcd* lines were higher than that in wild type (Figure 5C,D), and the results of DAB and NBT were consistent with the above-described results (Figure 5E,F). Thus, it is speculated that H_2_S may alleviate Mn stress in *Arabidopsis* seedlings by reducing reactive oxygen species content.

### 2.6. Effects on Antioxidant Enzyme Activity of lcd and OELCD under Mn Stress

**Figure 6:**
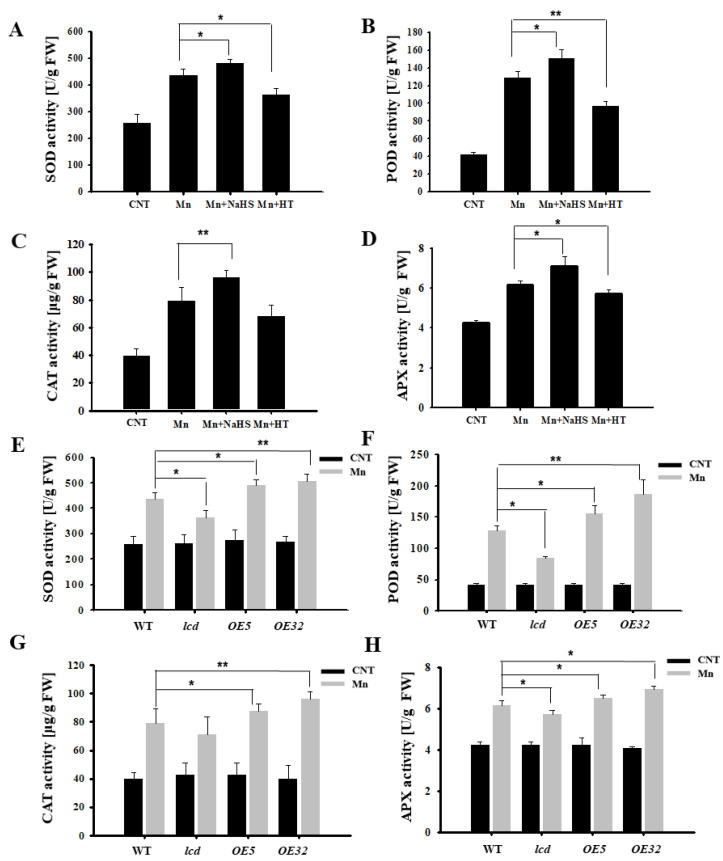
Effects of H_2_S on antioxidant enzyme activities in *Arabidopsis* seedlings under Mn stress. Effects of NaHS and HT on the activity of SOD (**A**), POD (**B**), CAT (**C**), and APX (**D**) in wild-type *Arabidopsis* seedlings under Mn stress. The activity of SOD (**E**), POD (**F**), CAT (**G**), and APX (**H**) in wild type, *lcd*, and two independent overexpression line seedlings treated with and without manganese. Three independent experimental replications were conducted. Values are the means ± SE of three independent experiments (* *p* < 0.05; ** *p* < 0.01).

Figure 5 shows that Mn stress increased the content of ROS in *Arabidopsis*, so the antioxidant enzyme activity was further examined. From Figure 6A–D, it can be deduced that the SOD, POD, CAT, and APX activities of *Arabidopsis* under 4 mM Mn^2+^ treatment were significantly higher than those of control. Additionally, the antioxidant enzyme activity of *Arabidopsis* with Mn treatment was upregulated significantly after NaHS treatment, whereas the activity of the antioxidant enzyme decreased after HT application. Additional results found that antioxidant enzyme activity of overexpression lines was significantly higher than that in wild type, and deletion mutants were significantly lower than that in the wild type under Mn^2+^ treatment (Figure 6E–H). It is, therefore, suggested that H_2_S may alleviate Mn stress in *Arabidopsis* seedlings by increasing antioxidant enzyme activity.

## 3. Discussion

It has been reported that H_2_S participates in a variety of growth and development processes, as well as stress responses, in plants [24]. In this study, we found that Mn stress can induce increased H_2_S content, and we also examined the enzyme activity and gene expression changes of the key synthase AtLCD in the H_2_S synthesis pathway. We speculate that H_2_S may participate in the Mn stress response (Figure 1). Through pharmacological experiments, wild-type *Arabidopsis* were used to observe the phenotype under Mn stress by external application of NaHS and HT, and the results showed that H_2_S was involved in the Mn stress activities and could alleviate the phenotype of Mn stress (Figure 2). Based on the results shown in Figure 1, the change in H_2_S content induced by Mn stress is from the AtLCD pathway, so further genetic evidence was provided. In Figure 3, we used 4 mM Mn at first, but the difference in phenotypes was not obvious, and the seedlings showed poor growth. It is speculated that the seeds were sowed directly in the culture medium to observe phenotype. And the time of treatment was long, the concentration was too high (4 mM), resulting in considerable damage to them. therefore, the concentration treatment was reduced (2 mM). Using *lcd* and *OELCD* as materials with Mn^2+^, Mn^2+^ and NaHS, and Mn^2+^ and HT treatments, phenotypic observations demonstrated that H_2_S alleviated Mn stress, and *lcd* was more pronounced than the wild-type Mn stress phenotype (Figure 3), which provided more evidence for H_2_S participation in Mn stress response. However, current studies have found that there are many types of synthetic H_2_S pathways in plants [25], and different synthetic pathways may be involved in different biological processes. Therefore, a question arises: Are there other sources of H_2_S in *Arabidopsis* besides the AtLCD pathway under Mn stress? Further studies are needed.

During plant response to heavy metal stress, the cell membrane can prevent or reduce the entry of metal ions into the cell. Additionally, plants can leave Mn in subcellular compartments, such as vacuoles, endoplasmic reticulum, Golgi, and cell walls to resist the toxic effects [23,26]. In *Arabidopsis*, AtNramp1 of the Nramp family is the main Mn transporter that participates in Mn uptake. AtNramp1 is localized to the plasma membrane of the epidermal cells of the root tips, and the expression of *AtNramp1* is upregulated when Mn is deficient [27]. The transport protein of *Arabidopsis* vacuole cation exchanger (CAX) is mainly involved in the transport of Mn^2+^ to the vacuole. The T-DNA knockout mutant of *AtCAX2* has a lower content of Mn^2+^ in the vacuole than that of the wild type. Overexpression of *AtCAX2 in tobacco increases resistance to Mn toxicity by mediating Mn chelation into the vacuole [28]. Endoplasmic reticulum-localized Ca^2+^-ATPase (ECA1) is another transporter that is intended to reduce the concentration of Mn^2+^ in the cytoplasm, as ECA1 can pump Mn^2+^ from the cytoplasm into the endoplasmic reticulum. Under high Mn conditions, the Arabidopsis* mutant *eca1* exhibited severe Mn stress symptoms, and overexpression of *ECA1* could restore the growth of *eca1* mutants to normal [29]. Metal tolerance protein (MTP), which is a Mn transporter in the CDF family [30], is responsible for transporting Mn into the vacuole and Golgi bodies. AtMTP11 may be in the irregular compartment of the trans-Golgi body [9]. For this group, *AtMTP11* has the highest expression and is more resistant to Mn in plant overexpression of *AtMTP11* [31]. Thus, we detected the expression of Mn-transport-related genes in the above transporter. The results showed that the expression of Mn-uptake-related gene *AtNramp1 in the mutant lcd* line was higher than that in the wild type under Mn stress, while both the expression levels of *OE5* and *OE32* were significantly lower than that in wild type (Figure 4C). This suggests that H_2_S may have prevented the uptake of Mn^2+^ by root cells, in part, by inhibiting the expression of the *AtNramp1* gene. This result is consistent with those related to hematoxylin staining (Figure 4A) and Mn content (Figure 4B). However, the expression levels of *AtCAX2*, *AtMTP11*, and *AtECA1 in lcd* lines were lower than that of wild type, while those of *OE5* and *OE32* were significantly higher than that of wild type (Figure 4D,F), which suggests that H_2_S may alleviate Mn stress by promoting Mn^2+^ transport to organelles.

Heavy metal stress can lead to excessive buildup of ROS, which may cause oxidative damage to biomolecules in plants [32]. H_2_S is a reductive substance and can directly scavenge ROS [33]. Therefore, we examined the content of ROS and the activity of antioxidant enzymes after Mn^2+^ treatment, and the results showed that Mn stress induced excessive ROS in plants (Figure 5). Both endogenous and exogenous H_2_S alleviated Mn stress in *Arabidopsis* seedlings by reducing ROS and significantly increasing antioxidant enzyme activity (Figure 6). It has previously been found that exogenous H_2_S can alleviate the degree of peroxidation caused to rice by mercury, thus alleviating the stress on rice and improving rice resistance [34]. Some studies have found that H_2_S alleviates oxidative stress and ionic toxicity in the cadmium-induced *Arabidopsis* roots through the hydrogen sulfide–cysteine circulatory system, thereby increasing the tolerance to cadmium [35]. Previous studies have found that plants respond to heavy metal copper ions, and H_2_S alleviates the *Arabidopsis* copper oxide stress process through a circulatory system with cysteine [36]. More research is warranted on whether cysteine participates in H_2_S involvement with Mn stress.

As a signaling molecule, how does H_2_S signaling occur when plants are subjected to stress? In recent years, the molecular mechanism by which H_2_S mediates the protein cysteine residue process in plants and animals, i.e., S-sulfhydration in post-translational modification, has been found [8,37]. Ethylene-induced hydrogen sulfide negatively regulates ethylene biosynthesis by the S-sulfhydration of ACO in tomatoes under osmotic stress [38]. Recently, it has been found that the persulfidation of SnRK2.6/OST1, a key regulatory protein of stomatal closure by H_2_S, promotes the activity of SnRK2.6 and its interaction with transcription factors downstream of the ABA signal, to promote stomatal closure and inhibit stomatal opening and improve the drought resistance of plants [39]. This characteristic of H_2_S provides an effective theoretical basis for finding its downstream regulatory proteins. Is the effect of H_2_S on the activity of plant antioxidant enzymes due to the S-sulfhydration modification or through other action modes? These issues need to be further studied to understand the diverse mechanisms of plant responses to Mn stress.

## 4. Materials and Methods

### 4.1. Experimental Materials

For *Arabidopsis*, the Columbia (Col-0) ecotype was taken as the genetic background, and the T-DNA insertion mutant (SALK_082099, named *lcd*) of *AtLCD* was purchased from the American *Arabidopsis* Biological Resource Center (ABRC). *AtLCD* overexpressing *Arabidopsis* was named *OELCD* and included 2 lines (*OE5* and *OE32*).

### 4.2. Material Constructs, Cultivation, and Treatment

Full-length *AtLCD* (At3g62130) was obtained from the *Arabidopsis* Information Resource (TAIR). The cDNA fragment was amplified by PCR with the primers as follows: forward primer, 5′- CCCAAGCTTATGGAGGCGGGAGAGCG-3′ with restriction site *Xba* I (TaKaRa, Maebashi, Japan), reverse primer, 5′-GGGGTACCCTACAATGCAGGAAGGTTTTGAC-3′ with restriction site *Kpn* I (TaKaRa, Maebashi, Japan). Then, the cDNA fragment was inserted into the *p-Super1300* vector (containing 35S promoter and *GFP* reporter gene) between restriction sites *Xba* I and *Kpn* I (TaKaRa, Maebashi, Japan). The construct was confirmed by restriction digestion and sequence analysis and then was named p-Super1300-*AtLCD.* Subsequently, the construct was transformed into *Agrobacterium tumefaciens* strain GV3101. The flower dip method was used to transform the *Arabidopsis* [40]. Two independent lines (*OE5* and *OE32*) from the T3 generation were used in this research. T-DNA insertion mutant (*lcd*) was identified by the three-primer method. The primers sequence were as follows: LP, 5’-CACTTTTGCAAGCTTGGTTTC-3’; RP, 5’-TCAATCCAGTTGAATAAGCGC-3’; LBb1: 5’-GCCTTTTCAGAAATGGATAAATAGCCTTGCTTCCT-3’. The mutant *lcd* was homozygous.

The full-fledged seeds were placed in a dark treatment at 4 °C for 2 to 4 days to break the dormancy; then, the seeds were cultivated in 2% MGRL culture solution (pH = 5.8), and the incubator was put in a light environment, which was adjusted to 22 °C with a light–dark cycle of 16 h/8 h. After 7 days, the seedlings were treated with 4 mM MnCl_2_, which was added to the MGRL culture solution; then, the material was collected for 0, 3, 6, 9, and 12 h to detect H_2_S content, AtLCD enzymatic activity, and *AtLCD* gene expression.

For *Arabidopsis* culture in a solid medium, *Arabidopsis* seeds were treated with 10% NaClO for 10–15 min, after which the seeds were washed with aseptic water until there was no NaClO residue. The seeds were then placed at 4 °C for dark treatment for 2 to 4 days to break dormancy, sowed to a solid medium (pH = 5.8), and placed in a light incubator (22 °C, light–dark cycle 16 h/8 h).

Growth of wild-type *Arabidopsis* was allowed for 4–5 days in the solid medium (pH = 5.8); the seedlings were then moved to 1/2 MS solid medium, 1/2 MS solid medium with 4 mM MnCl_2_, 1/2 MS solid medium with 4 mM MnCl_2_ and 0.1 mM NaHS (Sigma, St. Louis, MO, USA), and 1/2 MS solid medium with 4 mM MnCl_2_ and 0.02 mM HT (Sigma, St. Louis, MO, USA). All seedlings continued to grow for 5 days and were sampled for phenotype observation, detection of the growth indicators, and H_2_S content.

Growth of *Arabidopsis* was allowed for 4–5 days in the solid medium (pH = 5.8). Wild-type, *lcd*, *OE*5, and *OE*32 *Arabidopsis* seeds were moved to 1/2 MS solid medium and 1/2 MS solid medium with 4 mM MnCl_2_. All seedlings continued to grow for 5 days and were sampled for the physiological index.

Wild-type, *lcd*, *OE*5, and *OE*32 *Arabidopsis* seeds (100 seedlings of each kind of *Arabidopsis* in each treatment) breaking dormancy were sowed on 1/2 MS solid medium, 1/2 MS solid medium containing 2 mM MnCl_2_, 1/2 MS solid medium containing 2 mM MnCl_2_ and 0.1 mM NaHS, and 1/2 MS solid medium containing 2 mM MnCl_2_ and 0.02 mM HT, and then the phenotypes were observed and photographed after two weeks of growth.

Wild-type, *lcd*, *OE5*, and *OE32 Arabidopsis* seeds were allowed for 4–5 days in the solid medium (pH = 5.8); the seedlings were moved to 1/2 MS solid medium with 4 mM MnCl_2_ for 5 days, the roots of *Arabidopsis* seedlings were sampled for Mn content and Mn-transporter-related gene expression detection.

### 4.3. Detection of the Growth Indicators

The main root length, which was the distance from the base of the main root to the root tip was measured. The whole seedling from the medium was dried using filter paper, and the fresh weight was measured. Then, the whole seedling was dried at 80 °C for 16 h, and the weight was measured. The determination of chlorophyll content was performed according to the method of Lichtenthaler [41].

### 4.4. Determination of H_2_S-Related Indicators

H_2_S content detection was performed according to the methylene blue method described in Li et al. [42]; the determination of AtLCD enzyme activity followed the method of Riemenschneider et al. [43].

### 4.5. Determination of Physiological Indicators

Determination of hydrogen peroxide and superoxide anion content was achieved using the method of Zhao et al. [44], and the determination of SOD, POD, and CAT activities were determined following He et al. [45]; lastly, nitrogen-blue tetrazolium (NBT) (Macklin, China) and 3, 3′-diaminobenzidine (DAB) (Macklin, Shanghai, China) staining followed Jiang et al. [46].

### 4.6. Hematoxylin Staining

Hematoxylin staining followed the method of Ownby (1993), with minor modifications [22]. *Arabidopsis* seedlings were treated with 4 mM MnCl_2_ for 24 h, and then the residual treatment solution was washed with deionized water. The roots of *Arabidopsis* seedlings were dyed in hematoxylin dye (0.2 g of hematoxylin and 0.02 g of potassium iodide, a constant volume of 0.1 L, and stored away from light) for 2 h. Then, the dye solution was washed with deionized water and observed by a microscope.

### 4.7. Detection of Mn Content

Mn content detection was performed using the method of inductively coupled plasma atomic emission spectrophotometry (ICP–MS), as described in Delhaize et al. [31].

### 4.8. RNA Extraction and qRT-PCR

Total RNA was extracted using a TRIzol reagent (Invitrogen, Waltham, MA, USA), following the manufacturer’s instructions, and the cDNA was obtained by reverse transcription using the M-MLV RT Kit (Promega, Madison, WI, USA). With β-actin as an internal reference, qRT-PCR was performed in the presence of SYBR green I (BioWhittaker Molecular Applications, Walkersville, MD, USA) in the amplification mixture, and the data were analyzed using a MyiQ Detection System. The qRT-PCR procedure included 95 °C for 5 min, 95 °C for 30 s, 58 °C for 30 s, and 72 °C for 30 s, for 40 cycles. Three replicates were run for each sample. qRT-PCR primers are shown in Table 1.

### 4.9. Statistical Methodology

Statistical analysis for all experiments was carried out using SPSS. Data were analyzed with independent *t*-tests (*p* < 0.05). All the values presented are means of replicates ± SE of three independent experiments.

## 5. Conclusions

In summary, H_2_S is involved in the response of *Arabidopsis* to Mn stress and may alleviate the inhibition of Mn stress on *Arabidopsis* seedling growth by reducing Mn^2+^ content, reducing reactive oxygen species content, and enhancing antioxidant enzyme activity. This study provides an important basis for further study of plant resistance to heavy metal stress.

## Figures and Tables

**Table 1:** Primer sequences of qRT-PCR.

Gene Name	Primers’ Sequences (5′-3′)
*AtActin*	FP: GGTAACATTGTGCTCAGTGG
	RP: CACGACCTTAATCTTCATGC
*AtLCD*	FP: TGTATGTGAGGAGGAGGC
	RP: GTTTCATACTGATGCTGCTC
*AtNramp1*	FP: GCTGGACAATATGTAATGCAGG
	RP: CACCGATGAGAGCAACAATTAG
*AtCAX2*	FP: GCCTCTTAAATGCTACATTCGG
	RP: TCCTTTGTCAAAGACTTGGTCT
*AtECA1*	FP: GTACACACACAGTAGCTTCATG
	RP: GTTTGAGTCGAACGAGAAAGTC
*AtMTP11*	FP: CAATACGGACATGGTCAATGAC
	RP: AATGAGAGCCAAATGTGTATGC

## Data Availability

Data are contained within the article or Supplementary Materials.

## Supplementary Materials

The following supporting information can be downloaded at: https://www.mdpi.com/article/10.3390/ijms23095046/s1.

## Abbreviation

APX	Ascorbate peroxidase
CAT	Catalase
DAB	3, 3′-diaminobenzidine
H2S	Hydrogen sulfide
L-cysteine desulfhydrase	LCD
NaHS	Sodium hydrosulfide
Mn	Manganese
NBT	Nitrogen-blue tetrazolium
POD	Peroxides
ROS	Reactive oxygen species
SOD	Super oxide dismutase
