# Supplementary material for: Hydrogen Sulfide Alleviates Manganese Stress in Arabidopsis
Source: Int J Mol Sci. 2022 May 2;23(9):5046. doi: 10.3390/ijms23095046 (PMC9101000; doi:10.3390/ijms23095046)
Supplement: Supplementary file 1 [file ijms-23-05046-s001.zip › ijms-1597068-supplementary.pdf]

Supplementary Materials:

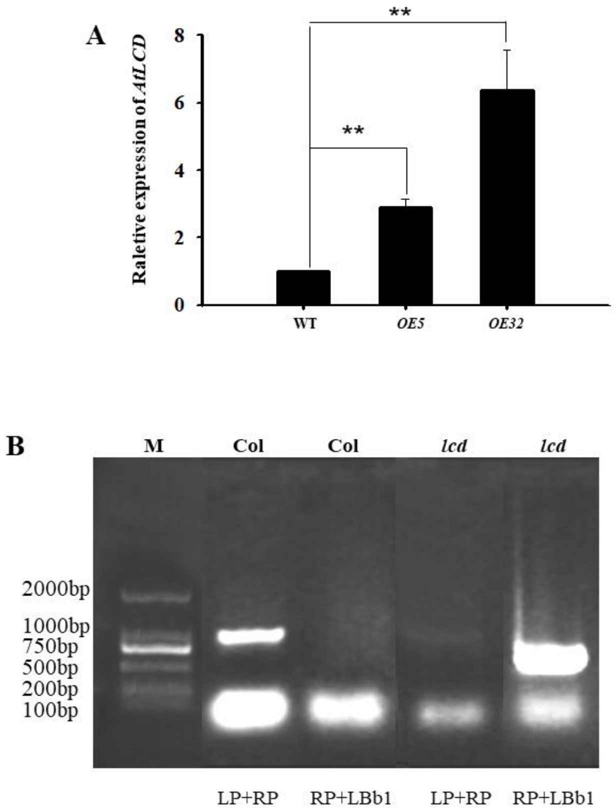

Figure S1. Identification of *lcd* mutant and *AtLCD* overexpressing lines. *AtLCD* relative expression in overexpressing lines (14 d- whole seedling of T3 generation lines) by qRT-PCR (A); Identification of *lcd* T-DNA-inserted mutant by PCR (B). Three independent experimental replications were conducted. Values are the means ± SE of three independent experiments (**$P <$ 0.01).

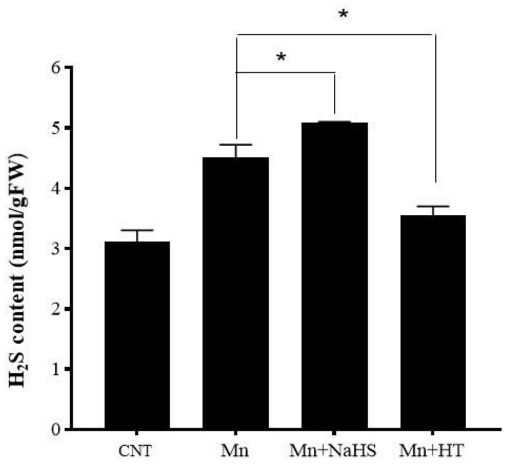

Figure S2. Effects of NaHS and HT on the content of H_2S in wild-type *Arabidopsis* seedlings under Mn stress. Three independent experimental replications were conducted. Values are the means ± SE of three independent experiments (*$P < 0.05$).
